# First Report on Microplastics Quantification in Poultry Chicken and Potential Human Health Risks in Pakistan

**DOI:** 10.3390/toxics11070612

**Published:** 2023-07-14

**Authors:** Muhammad Bilal, Madiha Taj, Habib Ul Hassan, Atif Yaqub, Muhammad Ishaq Ali Shah, Muhammad Sohail, Naseem Rafiq, Usman Atique, Mohammad Abbas, Saira Sultana, Umaiya Abdali, Takaomi Arai

**Affiliations:** 1 Department of Zoology, Government College University Lahore, Lahore 54000, Pakistan; 2 Department of Environmental Sciences, Government Degree College Gulabad, Adenzai 24461, Pakistan; 3 Department of Zoology (MRCC), University of Karachi, Karachi 75270, Pakistan; 4 Fisheries Development Board, Ministry of National Food Security and Research, Government of Pakistan, Islamabad 44000, Pakistan; 5 Department of Chemistry, Abdul Wali Khan University Mardan, Mardan 23200, Pakistan; 6 Department of Biology, Government Postgraduate College Sahiwal, Sahiwal 40210, Pakistan; 7 Department of Zoology, Abdul Wali Khan University Mardan, Mardan 23200, Pakistan; 8 Department of Bioscience and Biotechnology, College of Biological Systems, Chungnam National University, Daejeon 34134, Republic of Korea; 9 Department of Zoology, Quaid-i- Azam University, Islamabad, Islamabad 44000, Pakistan; 10 Dr. A. Q. Khan Institute of Biotechnology and Genetic Engineering, University of Karachi, Karachi 75270, Pakistan; 11 Environmental and Life Sciences Programme, Faculty of Science, Universiti Brunei Darussalam, Gadong BE 1410, Brunei

**Keywords:** microplastics, poultry, gastrointestinal tract crop, gizzard, polymers, colour, Pakistan

## Abstract

Microplastics (MPs) are an emerging environmental health concern due to their widespread occurrence in food sources such as fish, meat, chicken, honey, sugar, salt, tea and drinking water, thereby posing possible risks to human health. This study aimed to observe the existence of MPs in the crop and gizzard of the farm chicken, a significant food source in Pakistan. Twenty-four chicken samples were taken from eight poultry farms across Punjab, Pakistan. A total of 1227 MP particles were found from 24 samples (crop and gizzards) originating from the 8 poultry farms. In all, 429 MP particles were found in 24 chicken crops, with a mean of 17.8 ± 12.1 MPs/crop. In contrast, 798 MP particles were found in 24 chicken gizzards, with a mean of 33.25 ± 17.8 MPs/gizzard. Comparatively larger particles, ranging between 300–500 µm, were more abundant (63%) than other considered sizes (300–150 µm [21%] and 150–50 µm [16%]). Additionally, fragments were the dominant type of shape in both sample types (crop [64%] and gizzard [53%]). The predominant colours of particles extracted from gizzards and crops were yellow (32%) and red (32%), respectively. Chemical characterisation of these particles detected four types of polymers: polyvinyl chloride (PVC) at 51.2%, followed by low-density polyethylene (LDPE) at 30.7%, polystyrene (PS) at 13.6% and polypropylene homopolymer (PPH) at 4.5%. In conclusion, we provide evidence for MPs in the gizzards and crops of farmed chickens which may originate from contaminated poultry feed. Only a few studies have been reported globally to assess MPs ingestion in chickens. The current study is the first report from Pakistan. It could be a valuable addition to support MPs literature to establish a relationship between MPs contamination and intake through the food chain.

## 1. Introduction

Production of plastic polymers began in the middle of the 19th century and had exponential expansion in the 1950s [1]. Between 1950 and 2019, the yearly global production of plastic expanded from 1.5 million tonnes to 370 million tonnes, an almost 310-fold increase [2]. Due to their inherent non-biodegradability and ease of transmission by water and air currents, plastics are a pollution concern worldwide [1]. Due to their remarkable resilience and extended stability, most plastic products are not biodegradable and break down into small plastic particles called macroplastics, with a diameter > 5 mm, that can be easily distributed to other ecosystems by the action of wind and waves [3]. Nevertheless, the story does not end here, as these macroplastic particles further transform into smaller particles with a diameter < 5 mm, called microplastics, due to mechanical degradation, photodegradation or oxidation [4]. An updated definition of microplastics according to their size limit was presented by Koehler [5]: plastic particles larger than 5 mm are classified as macroplastics, those between 1 and 5 mm as mesoplastics, those between 0.1 µm and 1 mm as microplastics and those smaller than 0.1 µm as nanoplastics. Depending on the source, these microplastics can be classified as primary or secondary. Plastics used in cosmetics like microbeads and pellets, known as nurdles, which are raw materials for making plastic, are regarded as primary MPs because they are released into the environment in their micro size due to their manufacturing process [6,7]. Secondary MPs are produced when larger polymers undergo degradation due to various environmental causes [8,9,10]. Particle MPs have been divided into the following categories based on their shape geometry: fragment, film, pellet/granule, sheet, line/fibre and foam.

When MPs are introduced to an ecosystem, they bioaccumulate, circulate in the food chain and may negatively impact organisms [11,12]. If ingested, toxicological impacts may reduce chicken quality, which is of considerable attention for humans who eat chicken as a significant part of their meals. Numerous studies have documented the diverse toxicity of MPs in organisms, with altered behaviour, reduced survival and reproduction rates, a decline in body size, reduced motility, increased inflammation including intestinal defects, neurotoxicity, oxidative stress, genotoxicity and altered fat and energy metabolism being just a few of the significant adverse effects on animal development and health [13,14,15,16,17]. These are determined from laboratory exposures—often to extremely high levels of MPs. However, they signify that harm is possible if levels of MPs are allowed to increase in food and wildlife. Therefore, a precautionary approach is needed to ensure that food and the environment are plastic contamination-free.

Birds are used to evaluate the harmful impacts of environmental pollution since they are sensitive to and increasingly vulnerable to external environments. Hundreds of terrestrial and aquatic bird species have accumulated plastic waste and detritus, significantly increasing toxicity risk. Terrestrial birds have a variety of ecological roles in the food web, making them a crucial part of terrestrial ecosystems [18]. According to Zhao [19], MPs were found in the digestive systems of 16 out of 17 terrestrial bird species. The presence of macroplastic and microplastics have been observed in some raptors [18,20]. Since many seabirds eat aquatic species as their primary food source, they are frequently exposed to plastic when they consume aquatic species with bioaccumulated microplastics [21]. Up to 78% of seabird species have had MPs discovered in their digestive systems since the 1960s [22,23]. Furthermore, by 2050, nearly 99% of the more than 300 species of aquatic birds are anticipated to have eaten plastic particles [22].

According to research, consuming MPs is potentially harmful to birds’ reproductive systems [24,25]. For instance, Japanese quail chicks with observed plastic consumption had male epididymis intraepithelial cysts more frequently than those without plastic consumption [25]. Adult short-tailed shearwaters (*Ardenna tenuirostris*) can pass plastics or microplastics to their young, according to Carey [26]. The toxic effects of MPs in both chickens and humans are largely unknown. There is less research on terrestrial birds than aquatic ones; however, terrestrial birds play a vital role in the food web in the terrestrial ecosystem. Moreover, their study can evaluate and assess the environmental contamination of microplastics. Only a few studies report assessments on MPs ingestion in chickens around the globe. The current study is the first report from Pakistan. It could be a valuable addition to existing MP literature to establish a relationship between MPs’ pollution level and their intake through the food chain. It can also be significant to indicate potential sources of microplastics intake through consuming contaminated food in humans. Therefore, the present study aimed to highlight and report microplastic quantification from gizzards and crops of farmed chickens across Punjab, Pakistan.

## 2. Materials and Methods

### 2.1. Sample Collection and Chicken Dissection

In all, 24 chicken samples were collected from 8 different poultry farms in Punjab, Pakistan, with average age of 42.52 ± 3.8 days and weight of 1.6 ± 0.4 kg. All samples were killed and dissected immediately with no food administered outside the farms; crops and gizzards were transferred to pre-labelled beakers and then stored in a freezer for further analysis.

### 2.2. Sample Analysis

#### 2.2.1. Digestion

A 10% solution of potassium hydroxide (KOH), combined in a 5:1 ratio with the sample (*v*/*v*), was poured into the beaker containing sample matter for digestion and kept in a water bath at 55 °C for 36 h. KOH was recommended for digestion because it is reported to have little impact on microplastic degradation during digestion [27,28].

#### 2.2.2. Density Separation

The purpose of the density separation was to separate microplastic particles. After digestion, NaCl (3:1 *v*/*v*) was added and the mixture was stirred for 20 min before a 24 h settlement [29].

#### 2.2.3. Filtration

After the 24 h settlement, the supernatant layer of the sample was collected and passed through sieves of different pore sizes (500 µm, 300 µm, 150 µm and 50 µm) to obtain 3 different size fractions and each fraction was then filtered through filter paper using a filtration assembly. After filtration, the walls of the filtration assembly cup were washed twice and the filter paper containing solids was kept in a petri dish to dry for one day before detection [29].

#### 2.2.4. Microplastics Observation, Identification and Quantification

Inspections of dried filter papers containing particle content were carried out using a light binocular microscope (at 16 × 4 and 16 × 10 magnification, Labomed, model: CXL-110446002, 9135002, New York, NY, USA). Zeiss stereomicroscope stemi 508 microscopes at 2.5× magnification and a 1600X USB 8 LEDs electronic digital microscope camera were used to image identified particles. Any particle less than 50 µm was not measured in the current study. The physical shape, size, geometry and colour were considered for category identification. Polymer identification via FTIR spectroscopy (IRTracer-100, Shimadzu, Columbia, MD, USA) was carried out using the polymer spectral library of Omnic Spectra (Thermo Fisher Scientific Inc., Waltham, MA, USA) software.

### 2.3. Laboratory Contamination Control

All possible precautions were taken to prevent air contamination of samples. All laboratory equipment, including glassware and chemicals, was covered with aluminium foil when not used, because the samples were processed there. Reagents and distilled water were also filtered and wrapped in aluminium foil to protect them from environmental contamination. A few filter papers were positioned throughout the lab for 72 h in various positions to measure the suspended load of MPs from the environment. After that, these filter sheets were examined with a stereomicroscope. Six filter sheets and procedural blanks were reviewed during the analysis and kept as a control, no particles were detected in the procedural blanks.

### 2.4. Data Analysis

The data obtained during this study were subjected to analysis of variance (ANOVA) to compare the means.

## 3. Results and Discussion

### 3.1. Abundance of Extracted MPs

**Figure 1:**
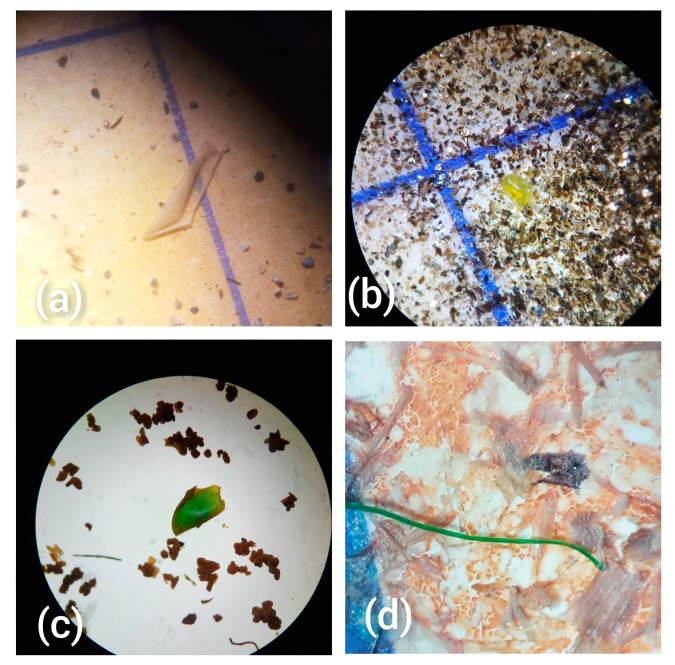
Microscopic images (at 16 × 4 and 16 × 10 magnification) of some of the extracted MPs representing different particle shapes: (**a**) sheet, (**b**,**c**) fragments and (**d**) fibres.

**Figure 2:**
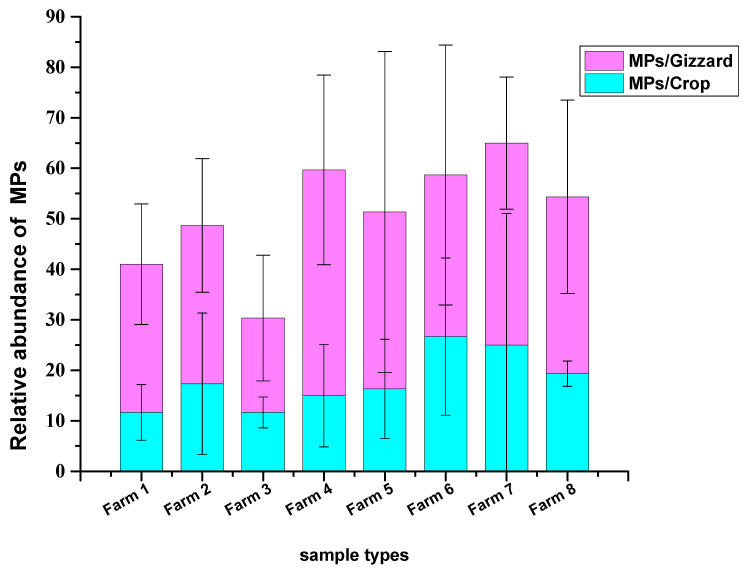
MPs number recovered from crops and gizzards of the chickens.

A total of 1227 particles were collected from 24 chicken samples (crop and gizzard for each) belonging to 8 different farms. A total of 429 MP particles were collected from 24 chickens crops, with a mean of 17.8 ± 12.1 MPs/crop. The highest abundance was detected in samples from farm 6, with a mean of 26.6 ± 15.5 MPs/crop, and the lowest concentrations were detected in farms 1 and 3 with mean values of 11.6 ± 5.5 MPs/crop and 11.6 ± 3.05 MPs/crop, respectively. A total of 798 MP particles were collected from 24 chicken gizzards with a mean of 33.25 ± 17.8 MPs/gizzard. The highest abundance of MPs was collected from the gizzards of farm 4, with a mean of 44.6 ± 18.7 MPs/gizzard, and the lowest from farm 3, with a mean of 18.6 ± 12.4 MPs/gizzard (Figure 1 and Figure 2). No statistically significant differences in the mean number of MPs recovered from the crop and gizzard were observed across all groups (*p* = 0.786, *p* = 0.842 for crop and gizzard, respectively).

Our findings for the microplastic content in crops and gizzards fall in the range of globally reported values such as Oliveri Conti et al. [30], who found 303 microplastic particles in 5 chickens with a mean of 60.5 MPs/bird. Huerta [31] extracted microplastics from a chicken’s gizzard (57 ± 41.1 MPs/gizzard) and crop (32.4 ± 15.1 MPs/crop). Another study reported MP particles in chicken gizzards with a concentration of 10.2 ± 13.8 MPs/g when no microplastic was found in crops and 45.82 ± 42.6 macroplastic particles per gizzard when they found 11 ± 15.3 macroplastic particles per crop [32]. Another study extracted 29 microplastic particles from 28 samples of birds’ gizzards [33]. In all, 643 particles were extracted from 44 gizzard samples from different bird species. The mean number of particles removed from each species was 29.9 ± 20.1 MPs/gizzard for brown pelicans, 7.6 ± 4.6 MPs/gizzard for laughing gulls and 9.6 ± 8.1 MPs/gizzard for double-crested cormorants [34].

Two bird species were analyzed by Deoniziak [35], where they extracted a total of 1073 MPs with a mean value of 31.56 ± 32.5 MPs per individual; 722 MPs were collected from gizzards of blackbirds and song thrushes. Collard et al. [36] collected 442 particles from 43 bird samples, with an average of 10.3 ± 1.8 MPs per individual, where the highest number of particles (198 particles out of 442) were found in gizzards (4.60 MPs/gizzard). Zhao et al. [19] found 364 particles in the gastrointestinal tract (GIT) of 16 birds, with a mean of 22.7/GIT. Apart from GIT, microplastic is also evident through inhalation in birds, such as a study by Takunaga [37], which found six microplastic particles in the lung tissue of wild birds from Japan. Three-hundred twenty particles/birds were extracted from GIT of the little black cormorant (*Phalacrocorax sulcirostris*) in Pulau Rambut Sanctuary, Indonesia [38]. Microplastics with an overall mean concentration of 11.9 ± 2.8 MPs/GIT were recovered from 8 species of birds in Florida, USA [18]. Generally, the concentration of MPs detected depends on the contamination of feeds, as these particles are potentially ingested through feed by the chickens (Table 1).

### 3.2. Size

**Figure 3:**
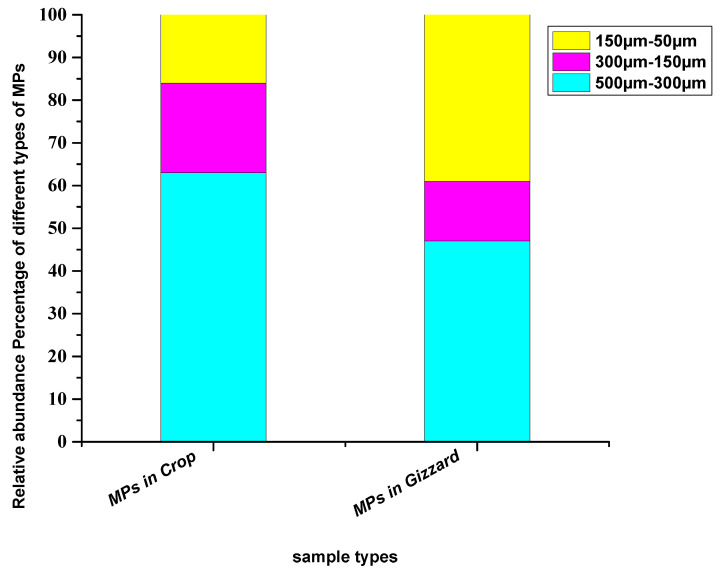
Relative abundance percentage of different sizes of particles extracted from chickens from all eight farms.

In terms of sizes of the particles extracted from crops, relatively larger particles (500–300 µm) were abundant with a percentage of 63%, followed by medium-size particles (300–150 µm) with a percentage of 21% and small particles (150–50 µm) with 16% of the total particles. In the gizzard, the detected particles were primarily large (500–300 µm) at 47%, followed by relatively smaller particles (150–50 µm) at 39%, while medium size (300–150 µm) was lowest at 14% (Figure 3). Different studies across the globe have reported an abundance of comparatively larger particles. Bessa [39] extracted 19 microplastic particles from the scat of penguins—majority of the identified particles were larger than 500 µm—where the mean size of the particles was 1266 ± 1378 µm. Liu et al. [40] have reported microplastics where the abundant particles were in the range of 500–1000 µm. Zhu et al. [41] collected particles from the GIT of birds with 92.9% consisting of particles > 5 mm and more than 90% were this size from Zhao et al. [19]. However, Deoniziak et al. [35] collected particles from the GIT of birds with sizes less than 1000 µm. A significant fraction (68.7%) of the extracted particles from GIT of the little black cormorant (*Phalacrocorax sulcirostris*) was in the range of 100–1000 μm [38] (Table 1). The potential reason for relatively larger particles may be that larger particles are less movable in the GIT tract and hence trapped particles sink in different parts of the GIT, including crops, gizzards and intestines. The smaller particles are more moveable through the GIT and tend to pass through faeces.

### 3.3. Shapes

**Figure 4:**
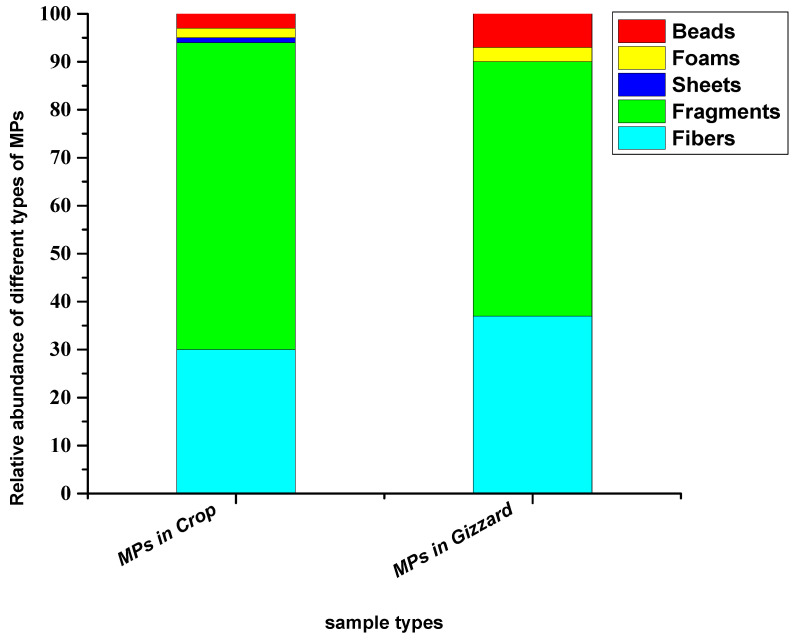
Relative abundance percentage of different types of shapes of particles extracted from chickens from all eight farms.

Based on the shape and geometry of the particles, five different types of shapes (fibres, fragments, foams, sheets and beads) were detected. Among these, fragments were the dominant type of shapes in both sample types (crop and gizzard). The abundance percentages of these different types of shapes in crops were, in decreasing order, fragments (64%), fibres (30%), sheets (3%), foams (2%) and beads (1%), while in gizzards they were fragments (53%), fibres (37%), sheets (7%) and foams (3%) (Figure 4).

Many studies have reported fragments to be the dominant shape of microplastic particles extracted from bird gizzards, such as Collard [42], who found 72.9% of particles as fragments. Takunaga [37] reported fragments as a dominant type of microplastics in their study on wild birds in Japan. Zhao [19] also reported that fragment is a significant type of particle shape of the extracted microplastics in their findings. The fragment percentage was 54.9% and the fibres were 37.4%. Unlike the current study, Deoniziak [35] reported fibre as a dominant shape (84%) of the particles in their finding and fragment as a minor fraction (10%). Weitzel [43] found fibres as a great shape in their results for seaside sparrows (*Ammospiza maritima*) in the Mississippi Gulf, where the percentage of fibre was noted to be 98%, followed by fragment (2%). Susanti [38] reported film as a dominant shape (75.0%) extracted from GIT of the little black cormorant (*Phalacrocorax sulcirostris*), followed by fibre (18.7%) and fragment (6.3%). Carlin [18] recovered microplastic particles from the GIT tract consisting of 86% fibres and 13% fragments, while beads were 0.3% in their findings (Table 1). The potential reason for dominance of fragment-type particles in crops and gizzards might be due to less mobility in the GIT tract and difficulty in excretion through faeces. Another possibility may be that chickens mistake the brightly coloured plastic pieces on the ground as food and selectively ingest them.

### 3.4. Colour

**Figure 5:**
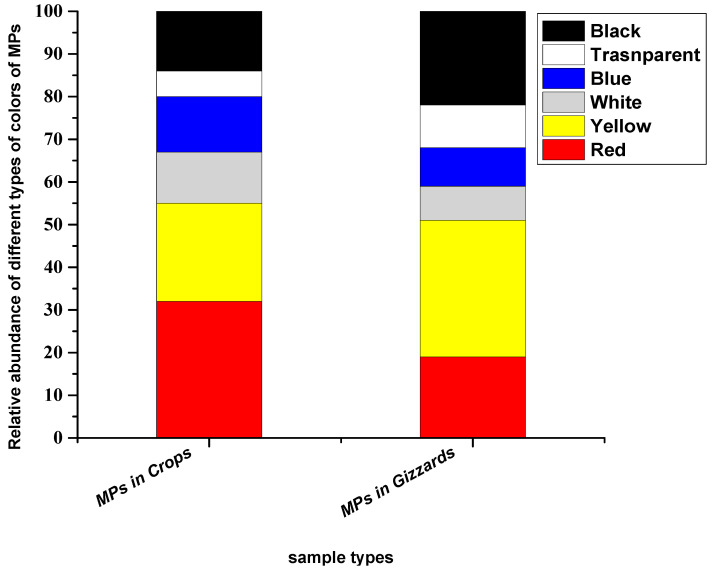
Colours of the MP particles recovered from chickens from all eight farms.

The particles extracted from crops and gizzards were noticed in six colours (red, yellow, white, blue, transparent and black). Among these, red colour was prominent in particles extracted from the crop, while yellow was dominant in particles extracted from gizzards. The abundance percentages of the particles in the crop were, in decreasing order, red (32%), yellow (23%), white (12%), blue (13%), transparent (6%) and black (14%), while in the gizzard, the percentages of different colours of the particles were red (19%), yellow (32%), white (8%), blue (9%), transparent (10%) and black (22%) (Figure 5).

Deoniziak [35] found transparent colour as dominant, followed by brown, with percentages of 74% and 14%, respectively. Another study [37] collected microplastics from the GIT of the little black cormorant (*Phalacrocorax sulcirostris*), where the dominant colours they noticed were transparent (56.2%), followed by red (18.7%) and black (12.5%), while blue and yellow colour were the same percentage (6.2%). Carlin [18] recovered microplastic particles from the GIT tract that were majority clear or royal blue. A study from Bustamante [33] reported that the most dominant colour of the extracted particles in the gizzards of Virginia waterfowl was blue (41.4%), followed by red (20.7%) and black (20.7%). These two dominant colours (red and yellow) may be due to their colourful nature and chickens mistake brightly coloured plastic pieces for food (Table 1).

### 3.5. Detected Polymer Types

**Figure 6:**
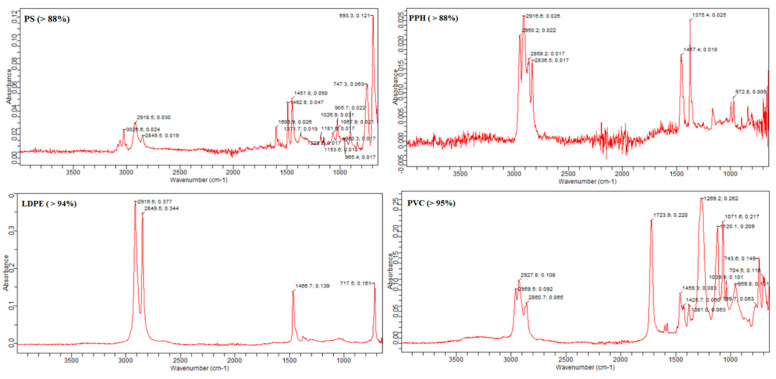
Detected polymer types in the current study.

For the chemical composition of the polymer type of these MPs, many studies have been carried out with Fourier transform infrared spectroscopy (FTIR). This is the most popular approach for chemical identification nowadays and works on a species-specific frequency absorbance of IR radiation. FTIR spectroscopy (IRTracer-100, Shimadzu, NYC, USA, Thermo Fisher Scientific Inc., USA software, New York City, NY, USA) made it possible to confirm the chemical composition of the kind of polymer [44]. The ATR sensor measured the MP particles and noted the absorbance peaks. Additionally, the peak similarity index was used to assess the composition of the particles by comparing recorded and standard peaks. Four types of polymer were detected: polyvinyl chloride (PVC), low-density polyethylene (LDPE), polystyrene (PS) and polypropylene homopolymer (PPH). The highest percentage of polymer detected was polyvinyl chloride (PVC) at 51.2%, followed by low-density polyethylene (LDPE) at 30.7%, while polystyrene (PS) was at 13.6% and polypropylene homopolymer (PPH) was at 4.5% (Figure 6). Different studies have reported different polymer types in their finding. However, some polymer types overlap throughout the globe in microplastic particles. Collard [42] also found that most of their microplastic particles were polypropylene, polystyrene and polyethylene. Among these, the dominant type of polymer was noted to be polyethylene. Another study has reported polyethylene terephthalate (16%), ethylene-co-polypropylene (11%) and cellulose, which was more abundant than the former two types of polymers, comprising 37% of the total particles [18]. Bessa [39] has identified different polymers in their study, including polypropylene, polyethylene, polyacrylonitrile and polyacrylate. These polymers were also mentioned as one of the primary forms of MPs in other research [44]. Most packaging includes foils, milk bottles, shampoo bottles, oil and soap bottles, while household items include trays, plates, cups, cables and PVC in electrical and electronic equipment. Tour tents and water pipes use LDPE and HDPE polymers [45]. PPH is used in packaging for several products, including structural tanks, battery covers and pump components [46] (Table 1).

### 3.6. Potential Human Health Risk

Human beings are the ultimate target of these MPs and NPs. Recently, many studies have evidently detected MP particles in human blood. Humans are exposed to inhaling or ingesting these microplastics or are exposed through the food chain because foodstuffs are contaminated with microplastic particles. Many reports have been published that quantify human materials, including blood, such as Leslie et al. [47], who have quantified nanoplastics with a size of 700 nm from human blood samples. In their study, the most abundant polymers they have identified in human blood are polyethylene, styrene and polyethylene terephthalate. Ragusa [48] found clear evidence of MPs in six human placentas taken from consenting women having healthy pregnancies and examined using Raman microspectroscopy to determine whether microplastics were present.

A study was designed to check for MPs larger than 50 µm in placental tissue and meconium samples taken during two breech births by cesarean section. The presence of 10 prevalent forms of microplastics was examined using Fourier transform infrared (FTIR) microspectroscopy in placenta and stool samples following the chemical digestion of non-plastic material. Their study found polyurethane, polyethylene, polypropylene and polystyrene [49]. Recently, MPs have been quantified in human breast milk; in their study, they extracted MPs from 26 out of 34 samples. The significant polymers extracted were PVC, PET and PP, with a size range of 2–12 μm [50].

Microplastics (MPs) are considered a global problem due to their toxicity in biota; however, their toxicity in chickens and humans is still unknown. This comprehensive study has the potential to add to existing knowledge regarding the ecotoxicity consequences of MPs in chickens and humans, which will be valuable for upcoming investigation. Swelling and blockages are caused due to the buildup of MPs and nanoplastics in tissues [51]. Microorganisms and pollutants have also been shown to be transported by these MPs and the intensity of exposure and sensitivity of the individual largely dictated adverse effects [52].

Oxidative stress has also been connected to MP exposure [53], cytotoxicity and spread to other tissues. In the ecosystem and among living things, MPs persist for a very long time. Since the animals are exposed to MPs for a long time, there is a chance that they will have chronic pain, swelling, cell proliferation and death, as well as immune cell impairment [54]. Patients with MPs had significantly greater inflammatory bowel disease rates than healthy individuals. Over time, PS MPs slowed the development of Caco-2 cells [55]. PS MPs, according to Wu et al. [55], perturbed the mitochondrial membrane potential. MPs could also act as vectors for bacteria [56]. They can absorb things from their environment or eject substances from their matrixes [4,57,58,59]. However, microplastic bioaccumulation and toxicity research, especially in humans, still needs improvement. There are few research reports, limited knowledge and little exploration due to ethical, social and other issues.

## 4. Conclusions

The present study found evident MP particles in the gizzard and crop of chickens; comparatively larger particles with a range of 300–500 µm were more abundant than other considered sizes of the particles (300–150 µm and 150–50 µm). Fragment-type particles were dominant based on the shape and geometry of the ingested particles. Collected particles were of six colours (red, yellow, white, blue, transparent and black), wherein the most particles were yellow and red extracted from gizzard and crops, respectively. Chemical characterisation of these particles has detected four types of polymers: polyvinyl chloride (PVC), low-density polyethylene (LDPE), polystyrene (PS) and polypropylene homopolymer (PPH). The highest percentage of polymer detected was polyvinyl chloride (PVC), followed by low-density polyethylene (LDPE). The present study showed that chickens we use as food sources contain MPs contamination and could be a potential risk for human consumption. The possible sources of this particle ingestion by the chickens are the feeds given and the farm’s environment. Future comprehensive studies are recommended to assess potential sources of microplastic intake in chicken and their transfer and bioaccumulation in the food chain.

## Figures and Tables

**Table 1:** Relative comparison of the current study to previous reports.

Particles	Region	Mean Number of MPs	Reference
	Pakistan	33.25 ± 17.8 MPs/gizzard, 17.8 ± 12.1 MPs/crop	Present Study
	Mexico	57 ± 41.1 MPs/gizzard, 32.4 ± 15.1 MPs/crop	[31]
	Mexico	45.82 ± 42.6 MPs/gizzard, 11 ± 15.3 MPs/crop	[32]
	Virginia, USA	1.03 MPs/gizzard	[33]
	Florida, USA	29.9 ± 20.1 MPs/gizzard	[34]
	Poland	31.56 ± 32.5 MPs/GIT	[35]
	Norway	4.60 MPs/gizzard	[36]
	Shanghai, China	22.7 MPs/GIT	[19]
	Indonesia	320 MPs/GIT	[38]
	Florida, USA	11.9 ± 2.8 MPs/GIT	[18]
	**Region**	**Dominant size range detected**	**Reference**
Size	Pakistan	300–500 µm	Present Study
	Antarctic region	>500 µm	[39]
	China	500–1000 µm	[40]
	South China	<5 mm	[41]
	Shanghai, China	>5 mm	[19]
	Poland	1000 µm	[35]
	Indonesia	100–1000 μm	[38]
	**Region**	**Dominant shape type detected**	**Reference**
Shape	Pakistan	Fragments (64%) in the gizzard Fragments (53%) in crop	Present Study
	Japan.	Fragments	[37]
	Norway	Fragments (72.9%)	[42]
	Shanghai, China	Fragments (54.9%)	[19]
	Poland	Fibres (84%)	[35]
	Mississippi Gulf	Fibres (98%)	[43]
	Indonesia	Film (75.0%)	[38]
	Florida, USA	Fibres (86%)	[18]
	**Region**	**Dominant colour detected**	**Reference**
Colour	Pakistan	Red (crop), yellow (gizzard)	Present study
	Indonesia	Transparent (56.2%)	[38]
	Florida, USA	Clear or royal blue	[18]
	Poland	Transparent (74%)	[35]
	Virginia, USA	Blue (41.4%)	[33]
	Norway	Yellow	[42]
	**Region**	**Polymer types detected**	**Reference**
Polymer	Pakistan	(PVC) with 51.2%, low-density polyethylene (LDPE) (30.7%), polystyrene (PS) (13.6%) and polypropylene homopolymer (PPH) (4.5%)	Present study
	Norway	Polypropylene, polystyrene and polyethylene	[42]
	Florida, USA	Polyethylene terephthalate (16%), ethylene-co-polypropylene (11%) and cellulose (37%)	[18]
	Antarctic region	Polyacrylonitrile, polypropylene, polyethylene and polyacrylate	[39]

## Data Availability

All data generated or analyzed during this study were included in this published article. All the raw and analyzed data will be available from the corresponding author based on reasonable demand.
